# Truth even unto its innermost parts

**DOI:** 10.7554/eLife.66850

**Published:** 2021-03-05

**Authors:** Eve Marder

**Affiliations:** Volen Center and Biology Department, Brandeis UniversityWalthamUnited States

**Keywords:** living science, public attitudes to science, disinformation, scientific integrity

## Abstract

Challenging anyone who spreads falsehoods is an important part of respecting the truth in both science and the wider world.

"Truth even unto its innermost parts" is the motto of Brandeis University where I have studied and worked for almost half a century. But those words could easily describe almost any university that brings together scholars and educators. The search for truth is central to the enterprise of philosophy, history, art, as well as for science. In fact, we take it as a given that all members of our community, regardless of how diverse their fields, share a common reverence for the search for truth. And a corollary of the search for truth must be our individual and collective willingness to admit mistakes, when evidence to that effect is brought to bear.

In contrast, in the United States (and in some other countries) we are living in an era in which people with different political and cultural points of view appear to live in alternate universes, and in which the concept of truth appears to be eschewed by many. However, many people who are willing to believe lies and falsehoods in the realm of politics also attempt to teach their children to always tell the truth. It is not that our population has lost its reverence for the concept of "truth": rather many seem to have lost their ability to distinguish between fact and fiction. This is not difficult to understand, as our collective national and international realities are not easily verified by our individual experience. Moreover, to the extent that our interactions with the world are mediated by electronic media, it appears quite difficult for many people to validate what they see and hear on social media and television.

One of the challenges of teaching science to young students is that we ask them to accept concepts and facts that are also outside of their direct personal experience. I mean, why should a high-school or university student believe us when we talk about neutrons or genome editing? Why should we expect students (or anyone else) to distinguish between the remarkable concepts and achievements of science and the many crackpot ideas that circulate on social media? What direct personal experience in their audience should scientists draw upon when trying to explain to the general public why they should give credence to medical experts and disavow anti-vaccinators? Why should we necessarily expect our public to understand the importance of masks and social distancing, if they know nothing about how viruses are transmitted, and why? For that matter, why should they link their own behavior to climate change when their direct experience is restricted to the weather and the price of energy? How do they know whom to believe?

When we speak to other scientists, we are often incredulous that the public comes to the conclusions that they sometimes do. But we shouldn’t be dismissive or surprised, because many of those conclusions are totally rational, given the direct and personal experiences of those who don’t take what we say as gospel according to science. Why should someone who has never had direct contact with modern science believe a scientist from an elite university or a faraway government laboratory over their own friends, family, neighbors, or clergy? If the scientific enterprise routinely boasts about breakthroughs in field of research as diverse as, say, stem cells and deep brain stimulation, should we be surprised that some people might believe that vaccination will implant a microchip that can control a person’s behavior?

Just as we know that a lie repeated many times is difficult to debunk in politics, scientific fictions and falsehoods can take on lives of their own if they are cited often enough. We reassure ourselves that the truth will always surface in science, but we underestimate the damage that can occur when a fallacious idea remains in circulation because those circulating the idea have not actually read and evaluated the primary papers that they are citing in support of the idea. We are all guilty of this, and we forget that many important ideas arose on the basis of evidence that we would today consider inadequate. Sometimes, prescient scientists saw the truth on the basis of partial evidence, luck, and terrific scientific intuition. Other times, very smart people, working with the tools and conceptual frameworks of their day, came to incomplete or incorrect conclusions.

**Figure 1:**
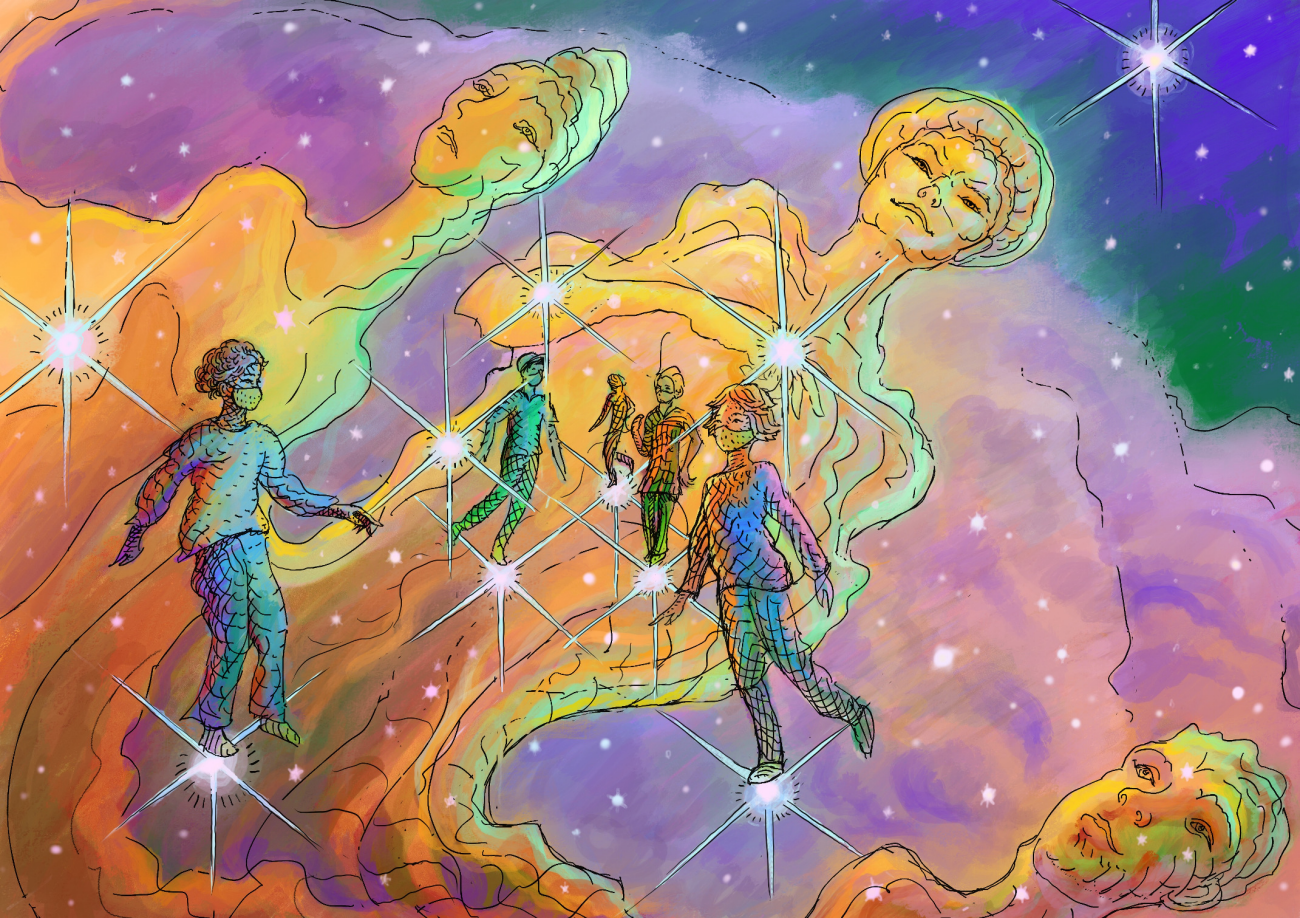
"Why should someone who has never had direct contact with modern science believe a scientist from an elite university or a faraway government laboratory over their own friends, family, neighbors, or clergy?".

Illustration: Ben Marder.

So even those of us who think that we are "following the evidence" may be guilty of some degree of carelessness and self-deception. The dramas of the flagrant disregard for data and truth that we see in today’s world should make us even more scrupulous about following the truth in our scientific lives. The temptations to oversell new results, and/or to underacknowledge the past accomplishments of others, are rampant, and are accentuated by the rapid pace and pressures of science today. But finding the truth does not come for free – it requires time and patience, which can be in short supply.

Those of us who are scientists have a special obligation to call out the truth, both in the context of the world and in our interactions with each other. But there are complex issues around telling the truth, collegiality, and politeness. Years ago I argued that senior investigators had an obligation to publically call out speakers at seminars and conferences if they were propagating fallacies or inaccuracies (Marder, 2003). This can be difficult to do if the speaker is a powerful senior figure, a young investigator, or a good friend. However, we need to signal to the audience that further data or thought might be required before the conclusions of a presentation can be taken at face value.

Moreover, being challenged in public can have an upside. A number of years ago, I was speaking about some work that Astrid Prinz, Dirk Bucher and I had just published on multiple solutions in neuronal networks (Prinz et al., 2004), and certain members of the audience argued (not always politely) that our conclusions would not hold if the networks were perturbed. After I heard this two or three times, I started seriously thinking about this issue, all of which triggered a major research program that has taken us in a number of new directions over the past fifteen years. This demonstrates, I would argue, that sometimes the biggest favor one can do for a colleague, friend, or mentee is to give them your unvarnished critiques of their new data and new ideas. Of course, the most gifted among us are able to be scientifically critical in remarkably constructive and positive ways. I aspire to the grace and generosity of such colleagues as I try to speak my mind in the service of logic and truth.

## Note

This essay is part of the Living Science collection.
